# Long-term body weight change assessed by non-contact load cells under the bed in older people with and without eating assistance: a preliminary study

**DOI:** 10.1038/s41598-022-12291-5

**Published:** 2022-05-16

**Authors:** Takahiro Ishikawa, Ikuko Sakai, Ayumi Amemiya, Ryou Komatsu, Shoko Sakuraba, Shiroh Isono

**Affiliations:** 1 Department of General Medical Science, Chiba University Graduate School of Medicine, Chiba, Japan; 2 Geriatric Medical Center, Chiba University Hospital, Chiba, Japan; 3 Graduate School of Nursing, Chiba University, Chiba, Japan; 4 Department of Anesthesiology, Graduate School of Medicine, Chiba University, Chiba, Japan

**Keywords:** Geriatrics, Weight management

## Abstract

Utilizing automatic daily body weight (BW) measurements may be helpful for assessing nutritional status and detecting underlying diseases particularly in older people who require nursing care. This preliminary study aimed to verify effectiveness of eating assistance for maintaining BW in older people using a contact-free load cells under the bed (Bed Sensor System: BSS). BW was measured every night for 3 months in eight nursing home older people with severe cognitive and physical dysfunctions. Body composition of the subject's trunk and each limb was measured using a segmented multi-frequency bioelectrical impedance analyzer (BIA). A monthly BW loss was estimated as a slope of linear regression of the daily BW plot. BSS successfully measured daily BW for the study period in all participants. The 4 residents with eating assistance gained slightly more weight, while the 4 residents without eating assistance lost weight. There was a significant difference between the two groups in the monthly BW change (− 0.79 ± 0.51 kg/month versus 0.20 ± 0.49 kg/month, P = 0.030). None of the BIA-derived parameters was associated with the monthly BW change. BSS revealed effectiveness of eating assistance to maintain BW in nursing home residents with severe cognitive and physical dysfunctions.

## Introduction

Weight loss is a bio-maker for malnutrition, frailty and the end of life in older people^1–3^. Older nursing home residents who need high levels of nursing home care are at higher risk of weight loss^4^. High-level care dependency and unexpected weight loss are the main factors for the decline of nutritional status in nursing home residents^5^. Eating assistance is commonly provided to older residents aiming for improving their nutritional status. In fact, by measuring body weight every month for a year in nursing home residents with low oral intake, eating assistance either twice per day during or between meals was demonstrated to successfully prevent weight loss^6^. Certainly, frequent body weight measurements serve to assess effectiveness of the eating assistance.

Despite importance of early detection of weight loss and intervention of eating assistance, frequent body weight measurements are difficult laborious task in nursing homes due to lack of staffs and limited physical functions of the older people. Because of the difficulties, little information is available regarding long-term body weight change and effectiveness of eating assistance in the nursing home residents with impairment of cognitive and physical performances which are common features of Japanese nursing home residents^7^. In this context, development of early and accurate diagnostic tools for weight loss without laborious burden to the nursing home staffs may improve nursing home care for older people.

We have developed a contact-free unconstraint vital sign monitor using high resolution strain gauge load cells placed under the bed legs (Bed Sensor Vital Sign Monitoring System: BSS, Minebea-Mitsumi, Nagano, Japan), allowing long-term continuous automatic measurements of body weight, time spending on bed, and vital signs including respiratory rate. We have already reported its accuracy for assessments of the respiratory status of healthy subjects^8^ and advanced cancer patients^9^. The purpose of this study is to test whether the BSS can be used in nursing home older patients to determine if mealtime assistance is effective in maintaining their weight.

## Methods

### Study design and participants

This observational study was approved by the Ethics Committee of the Graduate School of Nursing, Chiba University (Accreditation Number 29-27). The subjects of this study were older people living in a nursing home. After reviewing the cognitive and physical status of the residents with nursing staffs, we selected residents who are older than 75 years and require high levels of nursing care due to moderate to severe difficulties of physical and cognitive performance. The purposes and risks of this study were fully explained to the candidate residents and their families, and a written informed consent was obtained from each.

Body composition was routinely assessed at admission using a segmental multifrequency bioelectrical impedance analysis (BIA) device (InBody S10; Biospace, Koto-ku, Tokyo, Japan), which measured the body composition of the trunk and each limb separately in addition to a total body weight. Several BIA-derived parameters such as Skeletal Muscle Index, Body Fat, and Basal Metabolic Rate were obtained.

### Unconstraint under-bed vital sign monitoring for continuous body weight measurements

A contact-free unconstraint vital sign monitor using high resolution strain gauge load cells placed under the bed legs (Bed Sensor Vital Sign Monitoring System: BSS, Minebea-Mitsumi, Nagano, Japan) was used for continuous measurements of body weight and body movement parameters on the bed every minute. The BSS measurements were continued for more than 3 months without restricting routine care and daily activities. Among the BSS data collected during the nighttime hours from 2:00 a.m. to 3:00 a.m., body weight data with minimum body movements were selected and the values greater than one standard deviation were excluded as outliers. A single value of body weight for the day was determined as a median of the selected body weight data. With using the daily body weight data for the 3 months, monthly body weight loss was estimated as a slope of the linear regression line (kg/month).

### Data analysis

Based on the participants’ eating dependence, we compared the variables between participants with and without eating assistance (self-eating group: S-group versus eating assistance group: A-group). Variables were expressed as means and standard deviations. Comparisons between the groups were performed with independent t-test and Fisher’s exact test for continuous and categorical data, respectively (SigmaPlot 12.0; Systat Software Inc., Point Richmond, CA). A value of p < 0.05 was considered statistically significant, and all p-values were two sided.

### Ethics approval

The study protocol was approved by the Ethics Committee of the Graduate School of Nursing, Chiba University. All experiments were performed in accordance with relevant guidelines and regulations. Informed consent was obtained from all participants and/or their legal guardians. All Research have been performed in accordance with the Declaration of Helsinki.

## Results

### Background differences between the groups

Eight older residents participated in this study (Table 1). All participants needed high levels of nursing care either due to cognitive or physical dysfunctions while none had paralysis of their extremities. Based on eating dependency, four participants were classified into S-group and the other four participants were classified into A-group. Body weight, Body Mass Index, Skeletal Muscle Index and percentage of body fat did not differ between the two groups. Basal Metabolic Rate was slightly but significantly higher in S-group than A-group. Caloric intake tended to be higher in S-group.

**Table 1:** Participants’ background variables and results of body composition analysis.

Subject	Age (years)	Gender	Levels of care need	Need for eating assistance	Physical disability for daily life	Severity of dementia	Height (cm)	Body weight at admission (kg)	Body Mass Index (kg/m^2^)	Skeletal Muscle Index (kg/m^2^)	Body fat (%)	Basal metabolic rate (kcal/day)	Calorie intake (kcal/day)	Calorie intake (kcal/standard weight)	Calorie intake/basal metabolic rate
S1	79	Female	4	No	Moderate	Moderate	145.2	42.1	20.0	8.04	22.6	1087	1500	32.3	1.38
S2	86	Female	4	No	Mild	Moderate	145.5	37.4	17.7	7.56	11.8	1078	1400	30.1	1.30
S3	84	Female	4	No	Mild	Moderate	144.6	50.6	24.2	7.19	40.3	1018	1500	32.6	1.47
S4	88	Female	5	No	Moderate	Moderate	155.8	44.7	18.4	7.95	17.3	1183	1500	28.1	1.27
S group	84 ± 4						148 ± 5	43.7 ± 5.5	20.2 ± 2.9	7.68 ± 0.39	23.0 ± 12.3	1092 ± 68	1475 ± 50	31.0 ± 2.1	1.36 ± 0.09
A1	97	Female	5	Yes	Moderate	Severe	140.0	47.1	24.0	7.86	37.9	1038	1100	25.5	1.06
A2	95	Female	4	Yes	Moderate	Moderate	140.8	35.8	18.1	7.16	24.2	1013	1400	32.1	1.38
A3	87	Female	5	Yes	Moderate	Severe	147.3	41.3	19.0	6.71	26.1	1012	1200	25.1	1.19
A4	80	Male	5	Yes (PEG)	Severe	Mild	171.2	59.2	20.2	6.02	40.1	1118	1300	20.2	1.16
A group	90 ± 8						150 ± 15	45.9 ± 10.0	20.3 ± 2.6	6.94 ± 0.77	32.1 ± 8.1	1045 ± 50	1250 ± 129	25.7 ± 4.8	1.20 ± 0.13
P value	0.253	1.00	0.207				0.698	0.720	0.900	0.159	0.342	0.045	0.078	0.123	0.190

### Monthly body weight loss

**Figure 1:**
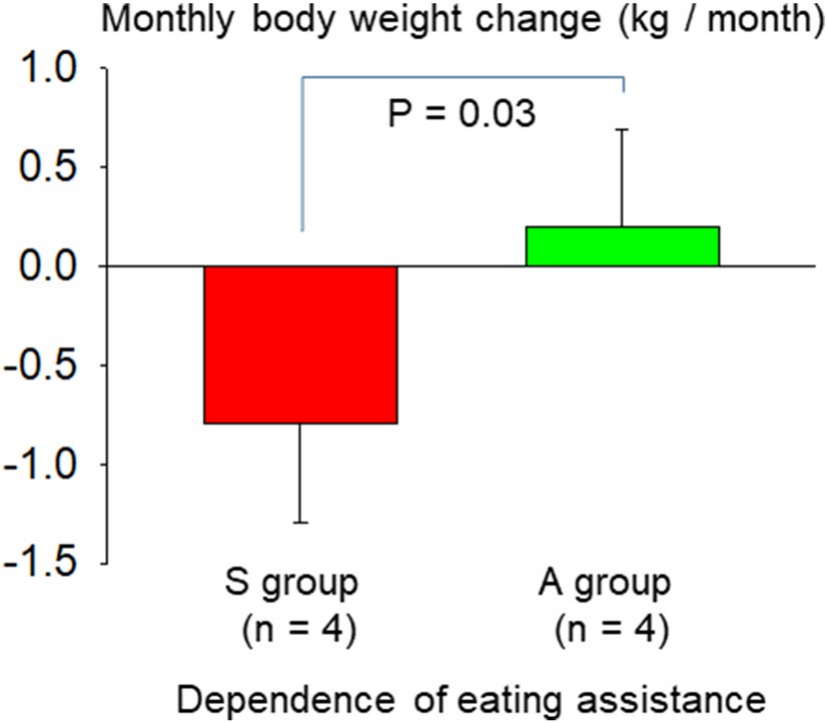
Comparison of monthly weight change between nursing home residents with (A-group) and without (S-group) eating assistance.

All 4 participants of S-group lost weight whereas 3 A-group participants did not lose weight during the 3-month study period. The A-group gained slightly more weight, while the S-group lost weight. There was a significant difference between the two groups in the monthly BW change (− 0.79 ± 0.51 kg/month versus 0.20 ± 0.49 kg/month, P = 0.030) (Fig. 1). None of the BIA-derived parameters was associated with the monthly body weight change.

## Discussion

In this preliminary observational study, we succeeded in automatic unconstrained daily body weight measurements for 3 months and estimated monthly body weight loss in nursing care residents with severe cognitive and physical dysfunctions. Monthly body weight loss was significantly greater in the residents without eating assistance than in those with eating assistance. Continuous body weight measurements with unconstraint under-bed vital sign monitoring may serve for assessing trends of nutritional status and general conditions in nursing home older people.

### Potential early recognition of weight loss with unconstraint under-bed vital sign monitoring for automatic daily body weight measurements

Weight loss has been reported to be an independent risk factor for death among older people in nursing homes^1,10^. A significant association between weight loss of 5 kg or more in a year and death within 6 months was documented^2^. Furthermore, one of the diagnostic criteria for frailty is a weight loss of 2–3 kg in 6 months^3^. Therefore, accurate diagnosis of weight loss requires long-term monthly body weight measurements as reported in many previous studies. However, such long-term diagnosis period may delay appropriate interventions for preventing development of malnutrition and frailty as the frailty-related diseases may have already progressed by the time of its diagnosis. Clearly, continuous assessments of monthly weight loss estimated by automatic daily body weight measurements used in this study are advantageous for early detection of frailty and potential end stage of the life over the conventional manual body weight measurements which are laborious and difficult in older people with physical disabilities. In fact, we identified three S-group participants and one A-group participant fulfilling the frailty criteria.

### Dietary intake methods and body weight loss

This is the first study assessing effectiveness of eating assistance for maintaining body weight of older nursing care residents with cognitive and physical dysfunctions. It has been previously reported that appropriate interventions for the elderly in nursing homes may improve weight loss in a comparison between groups requiring dietary assistance^11,12^. We found monthly body weight loss was significantly greater in the residents without eating assistance than in those with eating assistance. As discussed above, the weight loss detected in the residents without eating assistance fulfills the frailty criteria, and the potentially-pathological weight loss information could be used as an early alert for malnutrition. Previous studies have reported that delayed awareness of a gradual functional decline in dietary intake among nursing home staffs leads to weight loss and malnutrition in the residents, ultimately increasing the risk of death^13^. Despite importance of accurate dietary intake assessment, it is very difficult to objectively assess a gradual decline of the dietary intake. Furthermore, insufficient manpower in the nursing home, a common situation in Japan, was reported to be associated with weight loss of the residents^14^. Based on early recognition of weight loss by means of automatic daily body weight measurements, caregivers efficiently determine residents who really need eating assistance for recovering from the frailty process.

### Limitations of the study

There are several limitations to this study. First, the number of subjects in this study was small, which has insufficient statistical power. Also, there was low uniformity among the individual older. However, the analysis was performed after confirming that there were no significant differences between the S and A-group in terms of body composition using BIA, Calorie intake, and Calorie intake/Basal metabolic rate. Despite the limited number of subjects, it is interesting to note that the dietary assistance group maintained their weight significantly better than the independent diet group. In addition, it is very important to minimize the loss of lean body mass in the older people, but in this study, the observation period was short and it has not yet been possible to examine whether the suppression of weight loss by dietary assistance directly leads to the suppression of muscle mass loss. We plan to solve these problems by continuing this study in the future.

In conclusion, monthly body weight change can be estimated by automatic daily body weight measurements with unconstraint under-bed vital sign monitoring in nursing home residents with severe cognitive and physical dysfunctions. Eating assistance serves for maintaining body weight possibly preventing development of frailty.

## Data Availability

Under request to the corresponding author.
